# VEGF-A in COVID-19: a systematic review and meta-analytical approach to its prognostic value

**DOI:** 10.1007/s10238-025-01583-5

**Published:** 2025-03-12

**Authors:** Seyed Sobhan Bahreiny, Mohammad-Navid Bastani, Hossein Keyvani, Reza Mohammadpour Fard, Mojtaba Aghaei, Zahra Mansouri, Negin Karamali, Tannaz Sakhavarz, Mahdi Amraei, Elnaz Harooni

**Affiliations:** 1 Physiology Department, School of Medicine, Tehran University of Medical Sciences, Tehran, Iran; 2 Student Research Committee, Ahvaz Jundishapur University of Medical Sciences, Ahvaz, Iran; 3 Department of Medical Virology, School of Medicine, Ahvaz Jundishapur University of Medical Sciences, Ahvaz, 15794-61357 Iran; 4 Department of Virology, School of Medicine, Iran University of Medical Sciences, Tehran, Iran; 5 Department of Physiology, Physiology Research Center, Medical Basic Sciences Research Institute, School of Medicine, Ahvaz Jundishapur University of Medical Sciences, Ahvaz, Iran; 6 Department of Immunology, School of Medicine, Tabriz University of Medical Sciences, Tabriz, Iran; 7 Institute of Biochemistry and Biophysics (IBB), University of Tehran, Tehran, Iran

**Keywords:** Vascular endothelial growth factor, VEGF-A, COVI-19, Endothelial dysfunction SARS-CoV-2

## Abstract

Numerous studies have reported vascular endothelial growth factor A (VEGF-A) has a significant impact on the pathophysiology of COVID-19. The objective of this systematic review and meta-analysis is to determine the prognostic value of increased levels of VEGF-A in individuals with COVID-19. A systematic literature search was conducted across multiple electronic databases, including PubMed, Web of Science, Cochrane Library, Scopus, EMBASE, and Google Scholar, up to January 2024. Studies examining the levels of VEGF-A in the serum or plasma of COVID-19 patients were incorporated, with specific attention given to contrasting severe/critical cases against moderate cases. Standardized mean differences (SMD) with 95% confidence intervals (CIs) were calculated using a random-effects model to determine overall effect sizes. Meta-regressions and subgroup analyses were performed to explore potential sources of heterogeneity. The meta-analysis synthesized data from 11 studies involving a total of 1119 COVID-19 patients. Elevated levels of VEGF-A were significantly associated with disease severity, with a pooled SMD of 0.525 (95% CI 0.239–0.058; *P* = 0.028). Research has indicated that the nature of the relationship differs among various age groups, and there were minor discrepancies in the techniques employed to obtain VEGF-A measurements. Furthermore, meta-regression analysis indicated a potential correlation between VEGF-A levels and assay technique and body mass index (BMI). This meta-analysis provides compelling evidence for the prognostic potency of VEGF-A in COVID-19. Understanding the intricate interplay between VEGF-A and COVID-19 pathophysiology holds promise for the development of targeted therapeutic strategies and prognostic indicators in the management of COVID-19.

**Supplementary Information:**

The online version contains supplementary material available at 10.1007/s10238-025-01583-5.

## Introduction

The severe acute respiratory syndrome coronavirus 2 (SARS-CoV-2) is a potent pathogen within the Coronaviridae family and is recognized as the primary etiological factor responsible for the onset of coronavirus disease 2019 (COVID-19). The COVID-19 pandemic emerged in 2019 and rapidly escalated into a global health crisis, inflicting widespread devastation on communities and claiming millions of lives [1, 2]. This virus infects type II alveolar epithelial cells in the lungs [3, 4]. Although COVID-19 primarily affects the respiratory tract, abundant evidence shows that COVID-19 affects multiple organs and causes a wide range of clinical symptoms [3]. Within the realm of COVID-19, considerable focus has been directed toward the complex relationship involving vascular function and the severity of the condition. The severity of COVID-19 has been associated with vascular complications such as cardiac and renal failure, coagulopathy, pulmonary embolism, and the onset of acute respiratory distress syndrome (ARDS) [4, 5]. The development of ARDS is associated with substantial damage to both endothelial and epithelial cells. Consequently, the interaction between the virus and the endothelium could potentially lead to a systemic syndrome caused by COVID-19, characterized by endothelial and vascular dysfunction.

According to recent studies, COVID-19 patients experience interwoven angiogenesis in their lungs, which confirms the presence of endothelial damage [6, 7]. The concentration of vascular endothelial growth factor (VEGF) in the serum has emerged as a significant and potential biomarker choice. VEGF, which is recognized as an angiocrine factor, has attracted interest as a prognostic marker in the context of COVID-19 [8, 9]. Despite the observed correlation between serum VEGF levels and the severity of COVID-19, the exact mechanistic implications of VEGF on the pathophysiology of the disease remain unclear [10, 11]. Given these intriguing observations, this systematic review and meta-analysis attempts to determine the prognostic value of elevated VEGF in COVID-19 by summarizing existing evidence and analyzing the clinical relevance of serum VEGF levels in COVID-19 patients.

## Methods

### Registration and protocol

This review adheres to the Preferred Reporting Items for Systematic Reviews and Meta-Analyses (PRISMA) guidelines (updated in 2021) without significant deviations from the standard protocol. Furthermore, the systematic review has been duly registered in PROSPERO, an internationally recognized database for prospectively registered systematic reviews [CRD42023469722] [12, 13].

### Literature search & eligibility criteria

The search strategy was refined to encompass a broader scope by incorporating various synonyms and related terms. The following keywords and phrases were utilized: (“vascular endothelial growth factor” OR “VEGF” OR “VEGF-A”) AND (“COVID-19” OR “SARS-CoV-2” OR “coronavirus disease 2019” OR “novel coronavirus” OR “pandemic respiratory infection caused by SARS-CoV-2” OR “COVID-19 virus” OR “severe acute respiratory syndrome coronavirus 2” OR “2019-nCoV”). The search was conducted until January 2024 and included databases such as PubMed, Web of Science, Cochrane Central, EMBASE, and Scopus. A detailed explanation of the database search strategy is available in Supplemental Table 1. The inclusion criteria for studies comprised the following: (I) observational research studies that examined endothelial dysfunction biomarkers in COVID-19 patients aged 18 years and above, (II) studies primarily employing reverse transcriptase polymerase chain reaction for COVID-19 detection, (III) studies published in the English language, (IV) studies that reported soluble forms of biomarkers, (V) studies that compared clinical outcomes between COVID-19 patients with adverse and favorable outcomes, and (VI) studies that provided baseline values of the investigated biomarkers within the initial 72 h of hospital admission. Conversely, studies were excluded if they met any of the following criteria: (I) reviews, editorials, case reports, comments, guidelines, systematic reviews, and meta-analyses, (II) pre-print and unpublished studies, (III) studies lacking pertinent data, or (IV) studies that compared COVID-19 patients with individuals not affected by COVID-19. In order to enhance the comprehensiveness of the analysis, the reference lists of selected studies were scrutinized for additional relevant publications.

### Study selection

Two independent reviewers, S.B. and M.A., evaluated the eligibility of the studies using a systematic approach. Initially, they screened the titles and abstracts against predefined inclusion and exclusion criteria, which were developed in accordance with the study extraction framework outlined in our team’s prior research [14–18]. Following this preliminary screening, the reviewers conducted a detailed assessment of the full-text articles. Any disagreements between the two reviewers were resolved through discussion, and when necessary, by consulting a third reviewer to ensure consistency and accuracy.

### Data extraction

A comprehensive data extraction was carried out to collect all necessary information, which includes the names of authors, publication year, the number of patients, patient characteristics, study design, and baseline levels of the VEGF-A biomarker that was being investigated. The patients were categorized into two groups based on the severity of their disease and clinical outcomes. The first group consisted of patients with unfavorable outcomes, including those who were labeled as “severe,” “intensive care,” “critical,” “non-survivors,” “ICU patients,” “patients on mechanical ventilation (MV),” and “patients with thromboembolic events.” The second group comprised patients with favorable outcomes, including those described as “non-severe,” “non-ICU patients,” “inpatients,” “moderate,” “mild,” “noncritical,” “survivors,” “patients without MV,” and “patients without thromboembolic events” (Table 1).

**Table 1:** Characteristics of the studies included in the systematic review and meta‐analysis

Author, year [19]	Country	Study design	Number of participants	Poor outcome group *N* (%), age	Good outcome group N%, age	Severity outcome	Assay; sample	GRADE Assessment	NOS Score
Alfadda et al. [20]	Saudi Arabia	Prospective case–control	263	*N* = 202 (77%) 55.95 ± 17.00	*N* = 61 (23%) 45.95 ± 18.10	WHO criteria	Multiplex immunoassay; Serum	⊕ ⊕ ⊕ ⊕	7
Mescht et al. [21]	South Africa	Retrospective case–control	46	*N* = 37 (80.43%) 45.6 ± 11	*N* = 9 (19.56%) NA	Hospital admission	ELISA (R&D Systems); Plasma	⊕ ⊕ ⊕ ○	5
Tsuji et al. [22]	Japan	Prospective case–control	71	*N* = 25 (35.21%) 66 49–65	*N* = 46 (64.78%) 58.5 ± 49–69	Hospital admission	Multiplex immunoassay; Plasma	⊕ ⊕ ○○	8
Josuttis et al. [23]	Germany	Retrospective case–control	167	*N* = 139 (83.2%) 64.3 ± 18–94	*N* = 28 (16.7%) 63.7 ± 28–94	ICU admission	ELISA; Serum	⊕ ⊕ ○○	6
Tufa et al. [24]	Sweden	Observational cross-sectional	126	*N* = 68 (54%) 60 (22–86)	*N* = 68 (54%) 60 (22–86)	ICU admission	ELISA; (Meso Scale Diagnostics), Plasma	⊕ ⊕ ○○	6
Rodrigues et al. [25]	Germany	Retrospective case–control	23	*N* = 14 (61%) 61 (55–67)	*N* = 9 (39%) 64 (53–77)	Mechanical ventilation	Multiplex immunoassay; Serum	⊕ ⊕ ○○	7
Moti et al. [26]	Turkey	Prospective case–control	187	*N* = 95 (50.8%) 29.7 ± 7;	*N* = 92 (49.19%) 28 ± 7.75;	WHO criteria	ELISA; Serum	⊕ ⊕ ⊕ ○	8
Pine et al. [27]	USA	Retrospective case–control	49	N = 40 (82%) 62.1 ± 15	*N* = 9 (18%) 69 ± 21	ICU admission	ELISA (R&D Systems); Plasma	⊕ ⊕ ○○	7
Smadja et al. [28]	France	Observational cohort	40	*N* = 20 (50%) 59.5 (54.2–70.5)	*N* = 20 (50%) 53 (37–65.4)	ICU admission	ELISA (R&D Systems); Serum	⊕ ⊕ ⊕ ○	6
White et al. [29]	UK	Retrospective cross-sectional	109	*N* = 75 (69%) NA	*N* = 34 (31%) NA	WHO criteria	ELISA; Plasma	⊕ ⊕ ⊕ ○	8
Vassillou et al. [30]	Greece	Retrospective case–control	38	*N* = 10 (26%) 68 ± 10	*N* = 28 (74%) 62 ± 11	Mechanical ventilation	ELISA (R&D Systems); Plasma	⊕ ⊕ ⊕ ○	8

*GRADE* grading of recommendations assessment, development, and evaluation, *FF* follicular fluid. The : ⊕  ⊕  ⊕ ○ Moderate quality: We are moderately confident about the effect estimate; ⊕  ⊕ ○○ Low quality: Our confidence in the effect estimate is limited, ⊕ ○○○ Very low quality: We have very low confidence in the effect estimate

### Quality assessment

For the quality assessment of the studies included in our meta-analysis, we utilized the Newcastle–Ottawa Scale (NOS) for non-randomized studies. This scale is designed to assess the quality of observational studies with three broad criteria: selection of the study groups, comparability of the groups, and the ascertainment of either the exposure or outcome of interest (Supplemental Table 2).

**Table 2:** Meta-regression analysis of the included studies

Heterogeneity	Coefficient	95% CI		*P*_value_
Factors		LCI	*UCI*	
Publication year	0.346	0.095	0.598	0.106
Region	0.092	− 1.004	1.190	0.868
Total sample size	0.005	− 0.000	0.011	0.085
Study design	− 0.805	− 1.882	0.272	0.143
Assay technique	0.924	0.135	1.710	0.021
BMI	0.972	− 0.071	0.267	0.025
NOS score	− 0.033	− 0.537	0.471	0.008

*SE* standard error; *LCI* lower confidence interval; *UCI* upper confidence interval

Each study was awarded a star for each quality item appropriately addressed, with a maximum of nine stars. The quality of each study was categorized based on the number of stars: Studies with 0–3 stars were considered of low quality, 4–6 stars of moderate quality, and 7–9 stars of high quality. Studies of low quality were subjected to sensitivity analyses to determine the impact of their inclusion on the overall meta-analytical findings. Additional measures included a graphical exploration of funnel plots to assess publication bias and conducting sensitivity analyses to explore the influence of individual studies on the overall meta-analysis results. This robust approach ensured that our conclusions were based on data of the highest integrity and relevance to clinical and public health recommendations [31]. Furthermore, Table 1 represents the Grading of Recommendations Development, Assessment, and Evaluation technique (GRADE), which were employed to appraise the status and grade of the studies incorporated in this meta-analysis.

### Statistical analysis

The meta-analysis utilized Comprehensive Meta-Analysis (CMA) Version 4 software to facilitate statistical analyses. We employed standardized mean differences (SMD) ± standard deviation (SD) derived from baseline values of VEGF-A biomarkers to calculate pooled SMD and their corresponding 95% confidence intervals between COVID-19 patients with adverse and favorable outcomes [32, 33]. The heterogeneity among the studies was evaluated using the Chi-square Q test and the I-squared statistic. The I-squared statistic was then categorized as low (less than 25%), moderate (between 25 and 50%), or high (greater than 75%). We also planned to use a hierarchical summary receiver operating characteristic (H-SROC) analysis to evaluate the biomarkers’ sensitivity and specificity in predicting poor outcomes in COVID-19 patients. We also summarized the reported data for the area under the receiver operating curve, as well as the sensitivity and specificity of the studies included in our analysis. Subgroup analyses and meta-regressions were conducted to identify potential sources of variation in the data. Furthermore, the presence of publication bias was evaluated using the Egger and Begg tests, with a significance threshold of *P* < 0.05. The trim-and-fill method was used to estimate potentially missing studies due to publication bias in the funnel plot and to adjust the overall effect estimate.

## Results

### Selection process and characteristics of the included studies

**Fig. 1:**
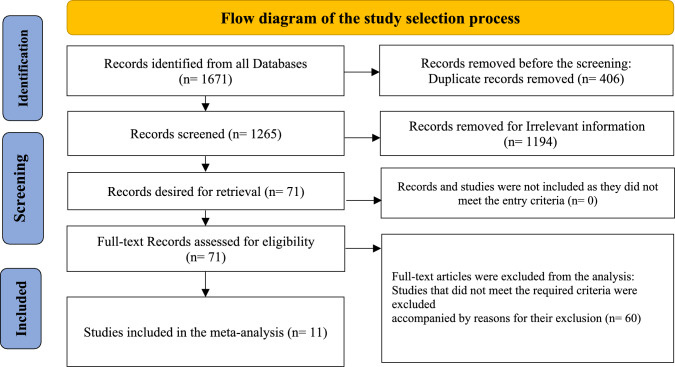
Flow diagram of study selection adjusted by PRISMA

The systematic search identified 1671 articles across various databases. After removing 406 duplicates, 1265 articles were reviewed by title and abstract. This screening led to the exclusion of 1194 articles that did not meet the eligibility criteria. The remaining 71 articles underwent full-text review, resulting in the exclusion of 60 for reasons such as lack of relevant outcomes related to VEGF-A levels, absence of a control group, or insufficient data on COVID-19 prognosis. Consequently, 11 studies were included in the meta-analysis, encompassing a total of 1119 participants (diagnosed with severe COVID-19 and 587 with mild to moderate COVID-19). The flow of study selection is illustrated in Fig. 1.

### Quality assessment

The quality scores of the studies included in the meta-analysis are presented in Supplemental Table 2. The NOS was utilized to evaluate the methodological quality of the included studies, with a maximum attainable score of 9. The included studies demonstrated variable quality, with scores ranging from 5 to 8. Specifically, studies such as Tsuji et al. [22] and Vassiliou et al. [30] received high-quality scores of 8, indicating robust methodology and comprehensive reporting. In contrast, Mescht et al. [21] scored 5, reflecting certain methodological limitations, including selection of controls and ascertainment of exposure.

Key domains of strength included adequate definition of cases and ascertainment of exposure, where most studies scored consistently high. However, variability was observed in the representativeness of cases and the same response rate across studies, potentially influencing the meta-analysis outcomes. These findings emphasize the need for consistent methodology in future research on VEGF-A and COVID-19 prognosis.

### Relationship between circulating VEGF-A levels and severity of COVID-19

#### Meta-analysis

**Fig. 2:**
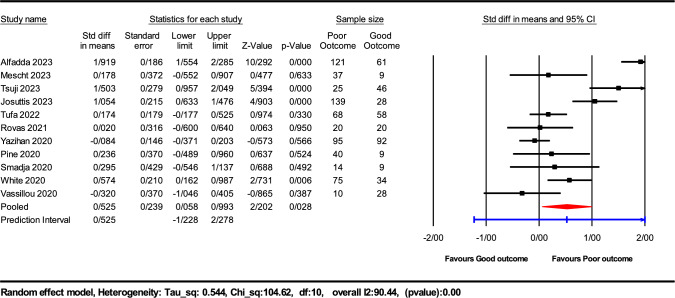
Forest plots illustrating the 11 assessable studies investigating VEGF levels in relation to COVID-19 outcomes

Our systematic review and meta-analysis assessed the prognostic potency of serum VEGF-A levels in COVID-19 patients, integrating data from 11 studies involving a total sample size of 1119 participants. The main meta-analysis revealed a statistically significant elevation in serum VEGF-A levels among patients with poor COVID-19 outcomes compared to those with favorable outcomes. The SMD was 0.525, with a 95% confidence interval (CI) ranging from − 1.22 to 2.27, and a *P* value of 0.028. A substantial degree of heterogeneity was observed among the included studies (I^2^ = 90.44%, *P* < 0.001), indicative of variability in study populations, methodologies, or both, as illustrated in Fig. 2.

#### Prediction interval

**Fig. 3:**
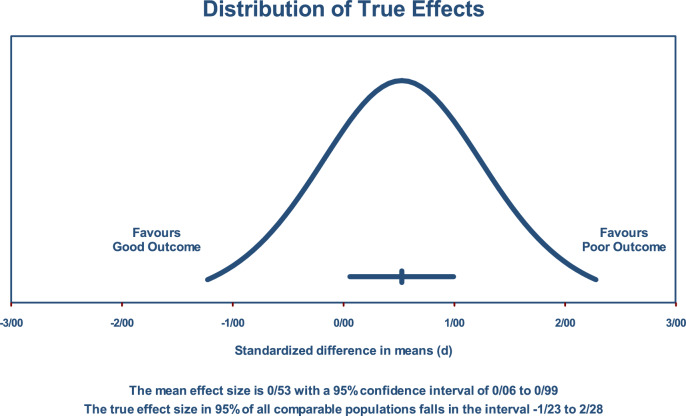
The prediction interval SMD of VEGF levels between COVID-19 outcomes groups

**Fig. 4:**
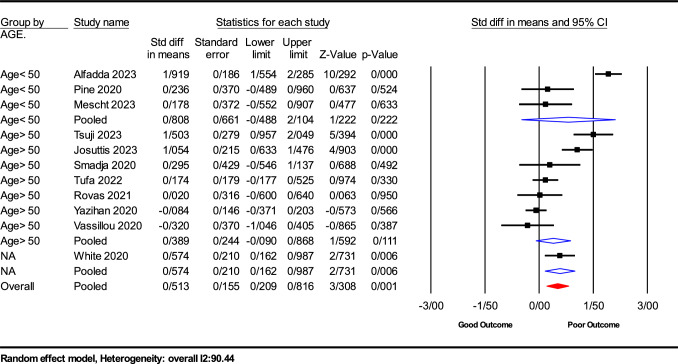
Subgroup analysis of VEGF levels by age IN COVID‐19 outcomes

**Fig. 5:**
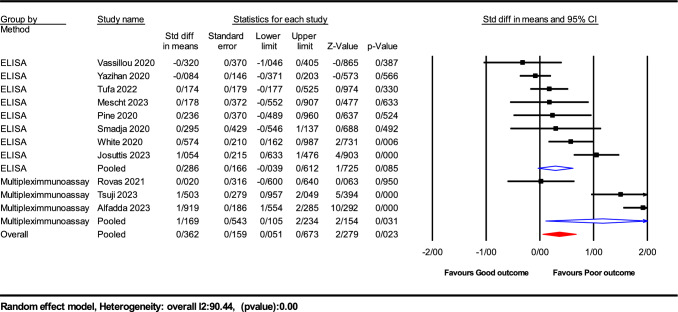
Subgroup analysis of VEGF levels by assay method in COVID‐19 Outcomes

**Fig. 6:**
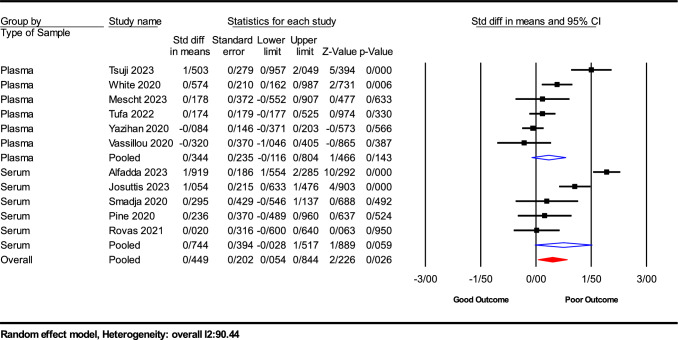
Subgroup analysis by sample type for VEGF Levels in COVID‐19 Outcomes

The estimated effect size followed a normal distribution, resulting in a prediction interval spanning from − 1.23 to 2.28. This prediction interval is designed to encompass the effect size within 95% of analogous populations while accounting for variables such as sample size and research methodology. It is imperative to exercise caution when interpreting findings within this prediction interval, as the true effect size may vary within this range. Figure 3 provides a visual representation of the prediction interval of their efficacy (Figs. 4, 5, and 6).

### Sensitivity analysis & publication bias

**Fig. 7:**
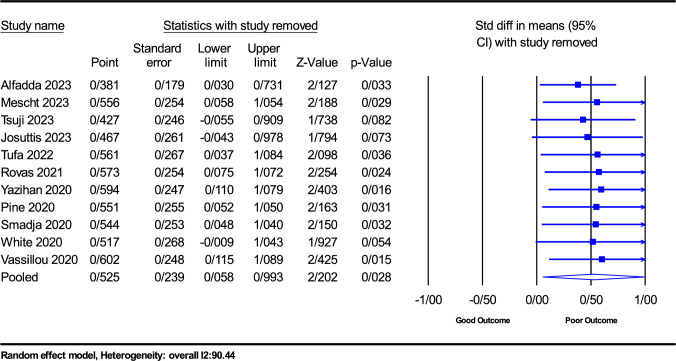
Sensitivity analysis was performed by excluding each study from the eligible studies

**Fig. 8:**
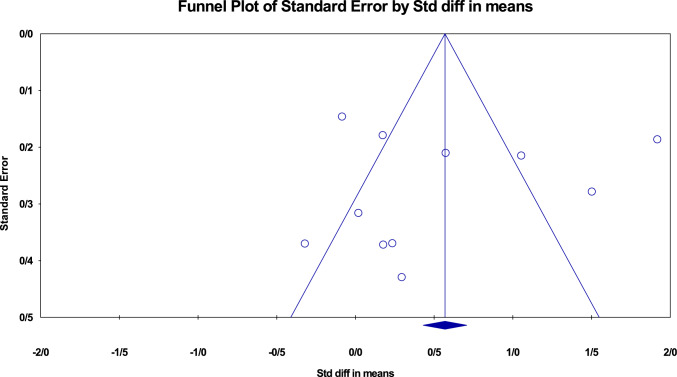
Funnel plot of standard error by standard differences in the means of serum VEGF level

A meticulous sensitivity analysis was conducted to assess the robustness of the current study by systematically excluding individual studies. Through this approach, the analysis revealed a range of SMD values, spanning from 0.239 to 0.058, concerning the sensitivity analysis of VEGF-A levels in COVID-19 patients compared to control groups. The lower limit of the 95% confidence interval was calculated at − 0.055 to 0.115, while the upper limit extended from 0.731 to 1.089. Importantly, these values maintained the clarity of I2 statistics. The sensitivity analysis reaffirmed the validity of the overall treatment effect (Fig. 7). The sensitivity analysis showed that the findings remained stable without any significant changes, which provided confidence in their robustness. Furthermore, the assessments conducted for publication bias using Egger’s test, along with the evaluation of funnel plot bias, as shown in Fig. 8, indicated a lack of substantial evidence for publication bias. Both the Egger test (*P* = 0.1423) and the Begg test (*P* = 0.1109) showed no publication bias. Furthermore, Supplemental Table 3 provides detailed results from the trim-and-fill method, which offers additional insights into bias evaluation.

### Subgroup analysis

Subgroup analysis was meticulously conducted to dissect the nuances in the relationship between serum VEGF-A levels and COVID-19 prognosis across various demographics and methodological approaches. The results, summarized in Fig. 4, revealed a differential impact of VEGF-A based on age, assay method, and sample type.

*Age-based analysis*: The analysis highlighted age as a significant modulator of VEGF-A levels. Patients 50 years and older exhibited a slight, though non-significant, increase in VEGF-A levels (SMD = 0.389; 95% CI − 0.090 to 0.868; *P* = 0.111). Conversely, younger individuals, specifically those below 25 years, showed a marginally higher elevation in VEGF-A levels compared to the control group, although this difference also did not reach statistical significance (SMD = 0.808; 95% CI − 0.488 to 2.104; *P* = 0.222).

*Assay method analysis*: The type of assay used for measuring VEGF-A levels significantly influenced the results. Subgroup analysis showed that patients analyzed using multiplex immunoassay techniques had notably higher levels of VEGF-A compared to those assessed with ELISA (SMD = 1.169; 95% CI 0.105–2.234; *P* = 0.031 for multiplex; SMD = 0.286; 95% CI − 0.039 to 0.612; *P* = 0.085 for ELISA).

Sample Type Analysis: The analysis differentiated between serum and plasma samples. Patients with serum samples showed no significant changes in VEGF-A levels (SMD = 0.744; 95% CI − 0.028 to 1.517; *P* = 0.059), whereas those with plasma samples exhibited a slight but not significant increase (SMD = 0.344; 95% CI − 0.116 to 0.804; *P* = 0.143).

### Meta-regression analysis

Meta-regression was employed to further explore the determinants influencing VEGF-A levels among COVID-19 patients, focusing on publication year, region, total sample size, test method, BMI, study design, and NOS score. This crucial information is comprehensively presented in Table 2.

Publication Year, Region, and Total Sample Size: These factors displayed a positive correlation with VEGF-A levels, though none were statistically significant, indicating a trend toward higher VEGF-A levels in more recent studies, larger studies, and certain geographical areas.

Assay technique and BMI: Both factors showed a significant positive correlation with VEGF-A levels. Higher BMI was associated with increased VEGF-A levels (meta-regression coefficient: 0.972; 95% CI − 0.071 to 0.267; *P* = 0.025), possibly due to the inflammatory state induced by higher body fat content, which could exacerbate the immune response to COVID-19. Similarly, more sensitive test methods correlated with higher detected levels of VEGF-A (meta-regression coefficient: 0.924; 95% CI 0.135–1.710; *P* = 0.021), underlining the role of assay sensitivity in capturing disease severity markers.

*Study design and NOS score*: Study design and Newcastle–Ottawa Scale (NOS) scores demonstrated a negative correlation with VEGF-A levels, suggesting that higher-quality studies or specific designs tend to report lower VEGF-A levels (study design regression coefficient = − 0.805; 95% CI − 1.882 to 0.272; *P* = 0.143; NOS score regression coefficient = − 0.033; 95% CI − 0.537 to 0.471; *P* = 0.008). This could be due to more stringent controls and sampling protocols in higher-quality studies, reducing potential biases and variability in VEGF-A measurement.

## Discussions

### Meta-analysis overview

This comprehensive meta-analysis investigates the prognostic potency of VEGF-A in patients with COVID-19, involving a diverse sample of 1119 participants across multiple studies. The findings consistently indicate a significant elevation in serum VEGF-A levels in patients with severe COVID-19 outcomes compared to those with milder symptoms. The implications of these results are profound, suggesting that VEGF-A could serve as a potential biomarker for COVID-19 prognosis and severity. This discussion elucidates the underlying biological mechanisms, interprets the primary findings, and explores their implications for clinical practice and future research.

### Interpretation of main findings

Our meta-analysis highlights a significant correlation between higher serum VEGF-A levels and poor outcomes in COVID-19 patients, with a standardized mean difference of 0.525. This suggests that VEGF-A may play a role in the pathogenesis or progression of severe COVID-19. The substantial heterogeneity observed (I^2^ = 90.44%) could be indicative of variability in the disease's impact based on demographic and clinical factors. Moreover, the prediction interval (− 1.23 to 2.28) underscores the variability in effect size, suggesting that while VEGF-A is generally predictive of poor outcomes, its specific utility might vary widely across different populations and clinical settings.

### Deciphering study outcome patterns: meta-regression insights

Meta-regression analysis explored various factors influencing VEGF-A levels, including publication year, region, and total sample size. While these factors showed a positive correlation, they were not statistically significant, indicating potential trends over time and geographical variations in VEGF-A expression possibly due to evolving laboratory techniques or regional differences in COVID-19 strain virulence [34, 35]. Notably, a significant positive correlation was found with BMI and test method. Higher BMI, associated with an inflammatory state, showed increased VEGF-A levels. This suggests that obesity-related inflammation might amplify the severity of COVID-19 via enhanced VEGF-A pathways, exacerbating the immune response [27, 36]. Additionally, more sensitive test methods were correlated with higher detected levels of VEGF-A, emphasizing the critical role of assay sensitivity in capturing disease severity markers effectively. Conversely, study design and NOS score displayed a negative correlation with VEGF-A levels. This finding suggests that higher-quality studies or specific designs, which likely incorporate more stringent controls and sampling protocols, tend to report lower VEGF-A levels. This could reduce potential biases and variability in VEGF-A measurement, leading to more accurate assessments of its role in COVID-19 prognosis [37, 38]. These findings are consistent with prior research on other biomarkers, reinforcing the broader applicability of such associations in disease prognosis and highlighting the necessity of rigorous methods and study quality for reliable biomarker analysis in disease prognosis [39–41].

### Prior research and contextualization

The current meta-analysis builds on a foundation of existing literature exploring the role of VEGF-A as a biomarker in various diseases, particularly in its well-established role in promoting angiogenesis and vascular permeability. Prior studies have also linked elevated VEGF-A levels with worse outcomes in respiratory diseases, such as ARDS, which shares some pathophysiological features with severe COVID-19, including hypoxia-induced vascular leakage and inflammation [42, 43]. Historically, research on VEGF-A in the context of infectious diseases has suggested that VEGF-A plays a significant role in the vascular and inflammatory responses to infections. For instance, increased VEGF-A levels have been observed in patients with H1N1 influenza and SARS, correlating with disease severity and outcomes [44, 45]. These precedents support the hypothesis that VEGF-A could be a critical mediator in the progression of COVID-19, particularly in its severe forms, where inflammatory and vascular complications dominate.

In the specific context of COVID-19, early studies have demonstrated that patients with severe outcomes exhibit significantly higher levels of VEGF-A compared to those with milder disease [23]. This correlation is thought to stem from role of VEGF-A in enhancing vascular permeability, thereby potentially exacerbating the pulmonary edema seen in severe COVID-19 cases [46]. VEGF-A interaction with immune modulation, specifically its effects on macrophage function and cytokine production, provides a plausible link to the cytokine storm syndrome frequently observed in severe COVID-19 patients [47].

Recent systematic reviews and meta-analyses prior to ours have provided mixed insights, with some studies indicating a strong prognostic value of elevated VEGF-A levels, while others pointed to a more nuanced role influenced by patient demographics, comorbidities, and disease severity. For example, a meta-analysis involving 7668 COVID-19 patients reported that while elevated VEGF-A levels were generally predictive of poor outcomes, significant heterogeneity across studies suggested that the prognostic utility of VEGF-A may depend on the population and local clinical practices [48]. This underscores the importance of context in interpreting VEGF-A levels, which our current analysis seeks to clarify further by including a more extensive and diverse dataset. These findings have profound implications, suggesting that VEGF-A holds promise as a potential target for therapeutic intervention. VEGF-A inhibitors, traditionally employed in oncology to impede tumor vasculature, could be repurposed to regulate the acute inflammatory response in COVID-19. Previous research has demonstrated their efficacy in mitigating the incidence or severity of complications such as ARDS and multiple organ failure [49–51]. However, given the pivotal role of VEGF-A in normal vascular function and wound healing, any therapeutic manipulation of its activity warrants cautious consideration.

### Biological mechanisms underlying roles of VEGF-A in COVID-19 severity

**Fig. 9:**
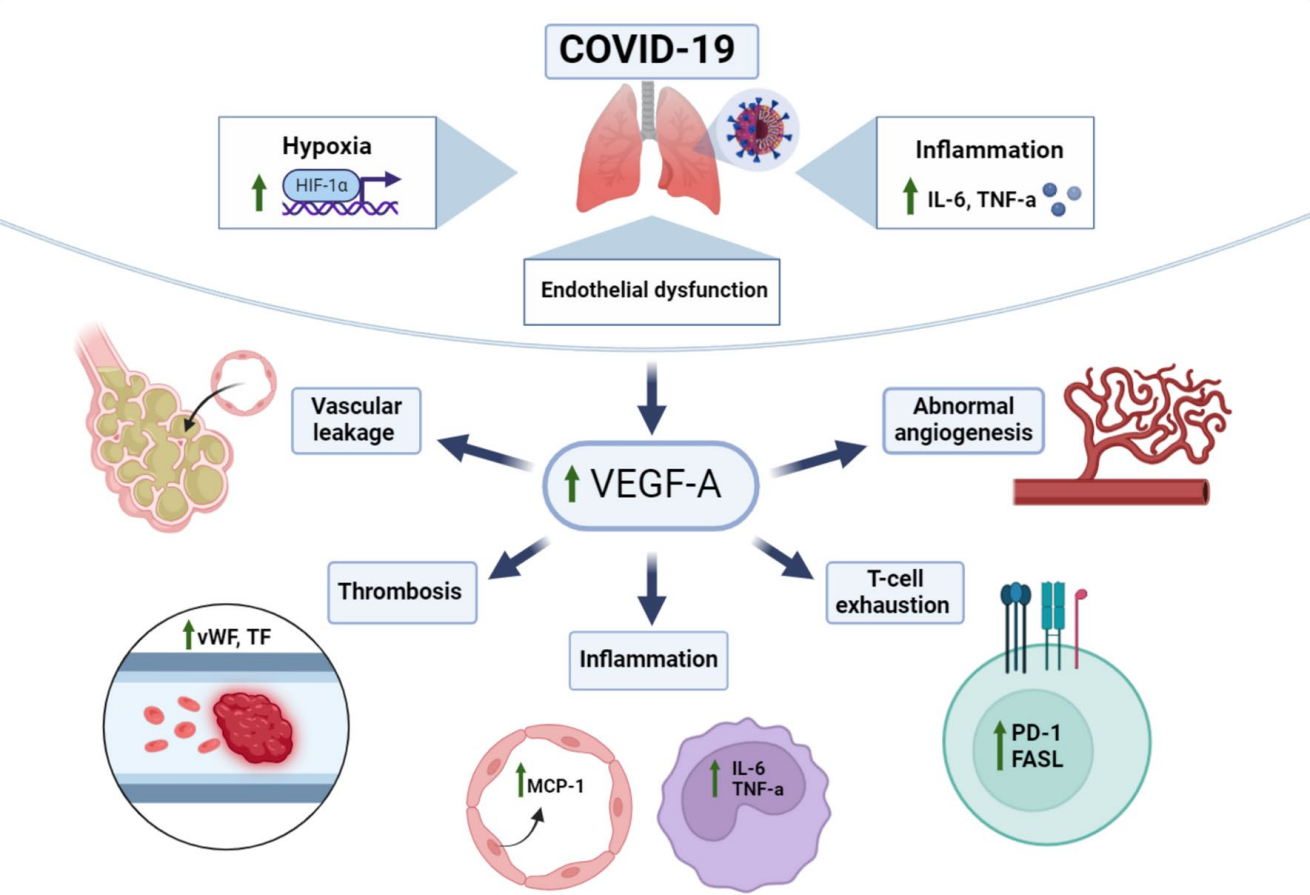
Graphical abstract illustrating the differential impact of VEGF-A on COVID-19 severity based on biological mechanisms

The underlying mechanisms connecting VEGF levels to the severity of COVID-19 involve both the factors that contribute to the elevation of VEGF in patients with COVID-19 and the subsequent impacts of heightened VEGF levels on the progression of the disease (Fig. 9).

VEGF-A is known to be upregulated in response to hypoxia, inflammation, and endothelial dysfunction, which are prominent features of severe COVID-19. Hypoxia, which results from impaired gas exchange in the lung due to viral-induced pneumonia, triggers the activation of hypoxia-inducible factor 1-alpha (HIF-1α) [52]. This transcription factor, in turn, stimulates the expression of VEGF-A to promote angiogenesis and improve tissue oxygenation [53]. In addition, the cytokine storms observed in severe COVID-19, characterized by elevated levels of interleukin-6 (IL-6) and tumor necrosis factor-alpha (TNF-α), directly stimulate VEGF-A secretion from activated endothelial and immune cells [54, 55]. Furthermore, the direct impact of the virus on endothelial cells in blood vessels can also increase VEGF-A expression. Endothelial dysfunction and injury trigger a compensatory response involving VEGF-A to promote angiogenesis and repair processes [56, 57].

On the other hand, the consequences of elevated VEGF-A levels in COVID-19 patients are multifaceted and can significantly influence disease severity and outcomes. In this context, one of the key mechanisms is promoting vascular permeability. Elevated VEGF-A levels disrupt endothelial cell junctions and increase fluid leakage into surrounding tissues [58, 59]. In patients with COVID-19, this vascular leakage leads to the development of pulmonary edema and ARDS, which are critical factors in disease progression toward severe respiratory failure [60]. Moreover, VEGF-A may exacerbate the hyperinflammatory response in COVID-19 patients by promoting immune cell recruitment and stimulating pro-inflammatory cytokine production [61–63]. This sustained inflammatory milieu contributes to the cytokine storm and multi-organ dysfunction observed in severe COVID-19 cases. Concurrently, VEGF-A can modulate adaptive immune responses in ways that impact viral clearance and disease resolution. Studies have shown elevated VEGF-A signaling is associated with T-cell exhaustion and dysfunction [64, 65]. This dysregulated immune response can worsen disease severity and increase susceptibility to secondary infections. VEGF-A is also implicated in regulating coagulation pathways. It is established that high VEGF-A levels can result in a hypercoagulable state by increasing the expression of tissue factor and the release of von Willebrand factor in endothelial cells [66, 67]. This effect can contribute to microvascular thrombosis and systemic coagulopathy, hallmarks of severe COVID-19. In addition, VEGF-A is a potent inducer of angiogenesis, the formation of new blood vessels. While this process is critical for tissue repair, excessive VEGF-A signaling can lead to aberrant angiogenesis and abnormal vascular remodeling. This may contribute to fibrotic changes in the lungs, potentially affecting long-term respiratory function in survivors [68]. In summary, elevated VEGF-A levels in COVID-19 patients reflect a complex interplay between viral infection, inflammation, hypoxia, and vascular dysfunction. This elevation contributes to the pathogenesis of severe disease manifestations, including ARDS, thrombosis, and immune dysregulation.

### Clinical implications and future directions

The significant correlation between elevated VEGF-A levels and worse COVID-19 outcomes suggests that VEGF-A could serve as a valuable prognostic biomarker for identifying patients at risk of developing severe disease. This could facilitate earlier, more targeted therapeutic interventions, potentially improving patient outcomes. Furthermore, understanding the mechanistic role of VEGF-A in COVID-19 could open new avenues for therapeutic strategies aimed at modulating this growth factor to mitigate disease severity.

Future research should focus on longitudinal studies to track VEGF-A levels over the course of COVID-19, from initial infection through recovery or progression to severe disease. Such studies could clarify the temporal dynamics of VEGF-A expression and its prognostic utility. Additionally, interventional studies examining the effects of therapies targeting VEGF-A pathways could provide insights into their potential benefits or risks in managing COVID-19.

### Strengths and limitations

This study boasts several strengths, including a meticulous search methodology, a substantial sample size drawn from multiple studies, and consistent findings that underscore the prognostic significance of VEGF-A within the COVID-19 landscape. The utilization of subgroup analyses and meta-regression techniques has enhanced the interpretation of results, thereby augmenting the relevance and generalizability of our findings.

However, several limitations warrant acknowledgment. Foremost among these is the variability in detection methods employed across the included studies, which may introduce discrepancies in reported VEGF-A levels. Despite efforts to standardize our analysis, variations in assay sensitivity, specificity, and calibration standards could potentially influence result consistency. Additionally, the evolving landscape of COVID-19 treatment modalities during the pandemic imposes further constraints on our analysis. Moreover, the influence of genetic factors, ethnicity, and geographical location on disease severity emerges as a significant area for future investigation. Furthermore, inconsistent reporting of therapeutic interventions, such as anticoagulants, steroids, and antiplatelets, is a significant limitation in our understanding of the impact of endothelial and angiogenesis biomarkers on COVID-19 prognosis. The potential effect of these medications on the observed associations between VEGF-A and COVID-19 prognosis is unclear, and further research is needed in this area.

## Conclusions

In conclusion, this systematic review and meta-analysis provides valuable insights into the prognostic role of elevated VEGF-A in COVID-19. While the precise mechanisms require further investigation, our findings of the present investigation indicate that VEGF-A levels could represent a valuable biomarker to determine COVID-19 patients at risk of poor outcomes [SMD: 0.525; *P* = 0.028]. This information could assist with early management and risk stratification, eventually improving patient care and outcomes in the midst of the current COVID-19 epidemic.

## Supplementary Information

Below is the link to the electronic supplementary material.

Supplementary file1 (DOCX 44 KB)

## Data Availability

No datasets were generated or analyzed during the current study.
