# Supplementary material for: VEGF-A in COVID-19: a systematic review and meta-analytical approach to its prognostic value
Source: Clin Exp Med. 2025 Mar 12;25(1):81. doi: 10.1007/s10238-025-01583-5 (PMC11903599; doi:10.1007/s10238-025-01583-5)
Supplement: Supplementary file 1 — Supplementary file1 (DOCX 44 KB) [file 10238_2025_1583_MOESM1_ESM.docx]

**Supplemental Table 1.** An overview of database search strategy.

| **Database** | **Query** | **Search Period** | **Results** |
| --- | --- | --- | --- |
| **Pub Med** | (((((((((((((((((((((((((((((((((((((Coronavirus*[Title/Abstract]) OR (COVID-19[Title/Abstract])) OR ("COVID 19"[Title/Abstract])) OR ((2019-nCoV[Title/Abstract]) AND (Infection*[Title/Abstract]))) OR ((SARS-CoV-2[Title/Abstract]) AND (Infection*[Title/Abstract]))) OR (("SARS CoV 2"[Title/Abstract]) AND (Infection*[Title/Abstract]))) OR ("2019 Novel Coronavirus Disease"[Title/Abstract])) OR ("2019 Novel Coronavirus Infection*"[Title/Abstract])) OR ((COVID-19[Title/Abstract]) AND ("Virus Infection*" [Title/Abstract]))) OR (("COVID 19"[Title/Abstract]) AND ("Virus Infection*"[Title/Abstract]))) OR (COVID19[Title/Abstract])) OR ((Coronavirus*[Title/Abstract]) AND ("Disease 2019"[Title/Abstract]))) OR ((Coronavirus*[Title/Abstract]) AND (Disease-19[Title/Abstract]))) OR ((Coronavirus*[Title/Abstract]) AND ("Disease 19"[Title/Abstract]))) OR ("Severe Acute Respiratory Syndrome Coronavirus 2 Infection*"[Title/Abstract])) OR (("COVID-19 Virus"[Title/Abstract]) AND (Disease*[Title/Abstract]))) OR ((COVID-19[Title/Abstract]) AND ("Virus Disease*"[Title/Abstract]))) OR ("SARS Coronavirus 2 Infection*" [Title/Abstract])) OR ((2019-nCoV[Title/Abstract]) AND (Disease*[Title/Abstract]))) OR (("2019 nCoV"[Title/Abstract]) AND (Disease*[Title/Abstract]))) OR ((COVID-19[Title/Abstract]) AND (Pandemic*[Title/Abstract]))) OR (("COVID 19"[Title/Abstract]) AND (Pandemic*[Title/Abstract]))) OR (SARS-CoV-2[Title/Abstract])) OR ("SARS Cov 2"[Title/Abstract])) OR ((SARS-CoV-2[Title/Abstract]) AND (Virus*[Title/Abstract]))) OR (("SARS CoV 2"[Title/Abstract]) AND (Virus*[Title/Abstract]))) OR (("2019 Novel"[Title/Abstract]) AND (Coronavirus*[Title/Abstract]))) OR (("Novel Coronavirus*"[Title/Abstract]) AND (2019[Title/Abstract]))) OR ((COVID-19[Title/Abstract]) AND (Virus*[Title/Abstract]))) OR (("COVID 19"[Title/Abstract]) AND (Virus*[Title/Abstract]))) OR ((Wuhan[Title/Abstract]) AND (Coronavirus*[Title/Abstract]))) OR ((COVID19[Title/Abstract]) AND (Virus*[Title/Abstract]))) OR ("Coronavirus Diease 2019 Virus*"[Title/Abstract])) OR ("Severe Acute Respiratory Syndrome Coronavirus 2"[Title/Abstract])) OR ((SARS[Title/Abstract]) AND ("Coronavirus 2"[Title/Abstract]))) OR (2019-nCoV[Title/Abstract])) OR ("Wuhan Seafood Market Pneumonia Virus"[Title/Abstract])) AND (((((((("Vascular Endothelial Growth Factor*"[Title/Abstract]) OR (VEGF*[Title/Abstract])) OR ("Vascular Endothelial Growth Factor A" [Title/Abstract])) OR ("Vascular Endothelial Growth Factor-A" [Title/Abstract])) OR (VEGF-A[Title/Abstract])) OR (Vasculotropin[Title/Abstract])) OR ("Vascular Permeability Factor*"[Title/Abstract])) OR ((Vascular[Title/Abstract]) AND ("Permeability Factor*"[Title/Abstract]))) | up to January 2024 | 450 |
| **Scopus** | ( ( ( TITLE-ABS-KEY ( coronavirus* ) ) OR ( TITLE-ABS-KEY ( covid-19 ) ) OR ( TITLE-ABS-KEY ( covid 19 ) ) OR ( ( TITLE-ABS-KEY ( 2019-ncov ) AND TITLE-ABS-KEY ( infection* ) ) ) OR ( ( TITLE-ABS-KEY ( sars-cov-2 ) AND TITLE-ABS-KEY ( infection* ) ) ) OR ( ( TITLE-ABS-KEY ( "sars cov 2" ) AND TITLE-ABS-KEY ( infection* ) ) ) OR ( TITLE-ABS-KEY ( "2019 novel coronavirus disease" ) ) OR ( TITLE-ABS-KEY ( "2019 novel coronavirus infection*" ) ) OR ( ( TITLE-ABS-KEY ( covid-19 ) AND TITLE-ABS-KEY ( "virus infection*" ) ) ) OR ( ( TITLE-ABS-KEY ( "covid 19" ) AND TITLE-ABS-KEY ( "virus infection*" ) ) ) ) OR ( ( ( TITLE-ABS-KEY ( sars-cov-2 ) AND TITLE-ABS-KEY ( virus* ) ) ) OR ( ( TITLE-ABS-KEY ( "sars cov 2" ) AND TITLE-ABS-KEY ( virus* ) ) ) OR ( ( TITLE-ABS-KEY ( "2019 novel" ) AND TITLE-ABS-KEY ( coronavirus* ) ) ) OR ( ( TITLE-ABS-KEY ( "novel coronavirus*" ) AND TITLE-ABS-KEY ( 2019 ) ) ) OR ( ( TITLE-ABS-KEY ( covid-19 ) AND TITLE-ABS-KEY ( virus* ) ) ) OR ( ( TITLE-ABS-KEY ( "covid 19" ) AND TITLE-ABS-KEY ( virus* ) ) ) OR ( ( TITLE-ABS-KEY ( wuhan ) AND TITLE-ABS-KEY ( coronavirus* ) ) ) OR ( ( TITLE-ABS-KEY ( covid19 ) AND TITLE-ABS-KEY ( virus* ) ) ) OR ( TITLE-ABS-KEY ( "coronavirus disease 2019 virus*" ) ) OR ( TITLE-ABS-KEY ( "severe acute respiratory syndrome coronavirus 2" ) ) OR ( ( TITLE-ABS-KEY ( sars ) AND TITLE-ABS-KEY ( "coronavirus 2" ) ) ) OR ( TITLE-ABS-KEY ( 2019-ncov ) ) OR ( TITLE-ABS-KEY ( "wuhan seafood market pneumonia virus" ) ) ) OR ( ( TITLE-ABS-KEY ( covid19 ) ) OR ( ( TITLE-ABS-KEY ( coronavirus* ) AND TITLE-ABS-KEY ( "disease 2019" ) ) ) OR ( ( TITLE-ABS-KEY ( coronavirus* ) AND TITLE-ABS-KEY ( disease-19 ) ) ) OR ( ( TITLE-ABS-KEY ( coronavirus* ) AND TITLE-ABS-KEY ( "disease 19" ) ) ) OR ( TITLE-ABS-KEY ( "severe acute respiratory syndrome coronavirus 2 infection*" ) ) OR ( ( TITLE-ABS-KEY ( "covid-19 virus" ) AND TITLE-ABS-KEY ( disease* ) ) ) OR ( ( TITLE-ABS-KEY ( covid-19 ) AND TITLE-ABS-KEY ( "virus disease*" ) ) ) OR ( TITLE-ABS-KEY ( "sars coronavirus 2 infection*" ) ) OR ( ( TITLE-ABS-KEY ( 2019-ncov ) AND TITLE-ABS-KEY ( disease* ) ) ) OR ( ( TITLE-ABS-KEY ( "2019 ncov" ) AND TITLE-ABS-KEY ( disease* ) ) ) OR ( ( TITLE-ABS-KEY ( covid-19 ) AND TITLE-ABS-KEY ( pandemic* ) ) ) OR ( ( TITLE-ABS-KEY ( "covid 19" ) AND TITLE-ABS-KEY ( pandemic* ) ) ) OR ( TITLE-ABS-KEY ( sars-cov-2 ) ) OR ( TITLE-ABS-KEY ( "sars cov 2" ) ) ) ) AND ( ( TITLE-ABS-KEY( "vascular endothelial growth factor*" ) ) OR ( TITLE-ABS-KEY ( vegf* ) ) OR ( TITLE-ABS-KEY ( "vascular endothelial growth factor a" ) ) OR ( TITLE-ABS-KEY ( "vascular endothelial growth factor-a" ) ) OR ( TITLE-ABS-KEY ( vegf-a ) ) OR ( TITLE-ABS-KEY ( vasculotropin ) ) OR ( TITLE-ABS-KEY ( "vascular permeability factor*" ) ) OR ( ( TITLE-ABS-KEY ( vascular ) AND TITLE-ABS-KEY ( "permeability factor*" ) ) ) ) | up to January 2024 | 1,365 |
| **Cochrane library** | (("Vascular Endothelial Growth Factor*"):ti,ab,kw OR (VEGF*):ti,ab,kw OR ("Vascular Endothelial Growth Factor A"):ti,ab,kw OR ("Vascular Endothelial Growth Factor-A"):ti,ab,kw OR (VEGF-A):ti,ab,kw OR ("Vascular Permeability Factor*"):ti,ab,kw OR ((Vascular):ti,ab,kw AND ("Permeability Factor*"):ti,ab,kw) OR (Vasculotropin):ti,ab,kw) AND ((Coronavirus*):ti,ab,kw OR (COVID-19):ti,ab,kw OR ("COVID 19"):ti,ab,kw OR ((2019-nCoV):ti,ab,kw AND (Infection*):ti,ab,kw) OR ((SARS-CoV-2):ti,ab,kw AND (Infection*):ti,ab,kw) OR (("SARS CoV 2"):ti,ab,kw AND (Infection*):ti,ab,kw) OR ("2019 Novel Coronavirus Disease"):ti,ab,kw OR ("2019 Novel Coronavirus Infection*"):ti,ab,kw OR (("COVID-19"):ti,ab,kw AND ("Virus Infection*"):ti,ab,kw) OR (("COVID 19"):ti,ab,kw AND ("Virus Infection*"):ti,ab,kw) OR ("COVID19"):ti,ab,kw OR ((Coronavirus*):ti,ab,kw AND ("Disease 2019"):ti,ab,kw              ) OR ((Coronavirus*):ti,ab,kw AND (Disease-19):ti,ab,kw) OR ((Coronavirus*):ti,ab,kw AND ("Disease 19"):ti,ab,kw) OR ("Severe Acute Respiratory Syndrome Coronavirus 2 Infection*"):ti,ab,kw OR (("COVID-19 Virus"):ti,ab,kw AND (Disease*):ti,ab,kw) OR ((COVID-19):ti,ab,kw AND ("Virus Disease*"):ti,ab,kw OR ("SARS Coronavirus 2 Infection*"):ti,ab,kw OR ((2019-nCoV):ti,ab,kw AND (Disease*):ti,ab,kw) OR (("2019 nCoV"):ti,ab,kw AND (Disease*):ti,ab,kw) OR ((COVID-19):ti,ab,kw AND (Pandemic*):ti,ab,kw) OR (("COVID 19"):ti,ab,kw AND (Pandemic*):ti,ab,kw) OR ("SARS-CoV-2"):ti,ab,kw OR ("SARS CoV 2"):ti,ab,kw OR (("SARS-CoV-2"):ti,ab,kw AND (Virus*):ti,ab,kw) OR (("SARS CoV 2"):ti,ab,kw AND (Virus*):ti,ab,kw) OR (("2019 Novel"):ti,ab,kw AND (Coronavirus*):ti,ab,kw) OR (("Novel Coronavirus*"):ti,ab,kw AND (2019):ti,ab,kw) OR ((COVID-19):ti,ab,kw AND (Virus*):ti,ab,kw) OR (("COVID 19"):ti,ab,kw AND (Virus*):ti,ab,kw) OR ((Wuhan):ti,ab,kw AND (Coronavirus*):ti,ab,kw) OR ((COVID19):ti,ab,kw AND (Virus*):ti,ab,kw) OR ("Coronavirus Disease 2019 Virus*"):ti,ab,kw OR ("Severe Acute Respiratory Syndrome Coronavirus 2"):ti,ab,kw OR ((SARS):ti,ab,kw AND ("Coronavirus 2"):ti,ab,kw) OR (2019-nCoV):ti,ab,kw OR ("Wuhan Seafood Market Pneumonia Virus"):tiab,kw) | up to January 2024 | 27 |
| **Web of Science** | (TS=(Coronavirus*) OR TS=(COVID-19) OR TS=("COVID 19") OR (TS=(2019-nCoV) AND TS=(Infection*)) OR (TS=(SARS-CoV-2) AND TS=(Infection*)) OR (TS=("SARS CoV 2") AND TS=(Infection*)) OR TS=("2019 Novel Coronavirus Disease") OR TS=("2019 Novel Coronavirus Infection") OR (TS=(COVID-19) AND TS=("Virus Infection*")) OR (TS=("COVID 19") AND TS=("Virus Infection*")) OR TS=(COVID19) OR (TS=(Coronavirus*) AND TS=("Disease 2019")) OR (TS=(Coronavirus*) AND TS=("Disease-19")) OR (TS=(Coronavirus*) AND TS=("Disease 19")) OR TS=("Severe Acute Respiratory Syndrome Coronavirus 2 Infection*") OR (TS=("COVID-19 Virus") AND TS=(Disease*)) OR (TS=("COVID-19") AND TS=("Virus Disease*")) OR TS=("SARS Coronavirus 2 Infection*") OR (TS=(2019-nCoV) AND TS=(Disease*)) OR (TS=(2019 nCoV) AND TS=(Disease*)) OR (TS=("COVID-19") AND TS=(Pandemic*)) OR (TS=("COVID 19") AND TS=(Pandemic*)) OR TS=(SARS-CoV-2) OR TS=("SARS CoV 2") OR (TS=(SARS-CoV-2) AND TS=(Virus*)) OR (TS=("SARS CoV 2") AND TS=(Virus*)) OR (TS=("2019 Novel") AND TS=(Coronavirus*)) OR (TS=("Novel Coronavirus*") AND TS=(2019)) OR (TS=(COVID-19) AND TS=(Virus*)) OR (TS=("COVID 19") AND TS=(Virus*)) OR (TS=(Wuhan) AND TS=(Coronavirus*)) OR (TS=(COVID19) AND TS=(Virus*)) OR TS=("Coronavirus Disease 2019 Virus*") OR TS=("Severe Acute Respiratory Syndrome Coronavirus 2") OR (TS=(SARS) AND TS=("Coronavirus 2")) OR TS=(2019-nCoV) OR TS=("Wuhan Seafood Market Pneumonia Virus")) AND (TS=(**"**Vascular Endothelial Growth Factor*"**)**OR TS**=(**VEGF***)**OR TS=(**"**Vascular Endothelial Growth Factor A**"**) OR TS=(**"**Vascular Endothelial Growth Factor-A**"**) OR TS=(**"**Vascular Endothelial Growth Factor*"**)**OR TS**=(**VEGF-A**)**OR TS=( Vasculotropin) OR TS=("Vascular Permeability Factor*") OR **(**TS**=(**Vascular**)**AND TS=(**"**Permeability Factor***"**V | up to January 2024 | 482 |
| **Embase** | (coronavirus*:ti,ab,kw OR 'covid 19':ti,ab,kw OR 'covid 19':ti,ab,kw OR ('2019 ncov':ti,ab,kw AND infection*:ti,ab,kw) OR ('sars cov 2':ti,ab,kw AND infection*:ti,ab,kw) OR ('sars cov 2':ti,ab,kw AND infection*:ti,ab,kw) OR '2019 novel coronavirus disease':ti,ab,kw OR '2019 novel coronavirus infection*':ti,ab,kw OR ('covid-19':ti,ab,kw AND 'virus infection*':ti,ab,kw) OR ('covid 19':ti,ab,kw AND 'virus infection*':ti,ab,kw) OR 'covid19':ti,ab,kw OR (coronavirus*:ti,ab,kw AND 'disease 2019':ti,ab,kw) OR (coronavirus*:ti,ab,kw AND 'disease 19':ti,ab,kw) OR (coronavirus*:ti,ab,kw AND 'disease 19':ti,ab,kw) OR 'severe acute respiratory syndrome coronavirus 2 infection*':ti,ab,kw OR ('covid-19 virus':ti,ab,kw AND disease*:ti,ab,kw) OR ('covid 19':ti,ab,kw AND 'virus disease*':ti,ab,kw) OR 'sars coronavirus 2 infection*':ti,ab,kw OR ('2019 ncov':ti,abkw AND disease*:ti,ab,kw) OR ('2019-ncov':ti,ab,kw AND disease*:ti,ab,kw) OR ('covid 19':ti,ab,kw AND pandemic*:ti,ab,kw) OR ('covid 19':ti,ab,kw AND pandemic*:ti,ab,kw) OR 'sars-cov-2':ti,ab,kw OR 'sars cov 2':ti,ab,kw OR ('sars-cov-2':ti,ab,kw AND virus*:ti,ab,kw) OR ('sars cov 2':ti,ab,kw AND virus*:ti,ab,kw) OR ('2019 novel':ti,ab,kw AND coronavirus*:ti,ab,kw) OR ('novel coronavirus*':ti,ab,kw AND 2019:ti,ab,kw) OR ('covid 19':ti,ab,kw AND virus*:ti,ab,kw) OR ('covid 19':ti,ab,kw AND virus*:ti,ab,kw) OR (wuhan:ti,ab,kw AND coronavirus*:ti,ab,kw) OR (covid19:ti,ab,kw AND virus*:ti,ab,kw) OR 'coronavirus disease 2019 virus*':ti,ab,kw OR 'severe acute respiratory syndrome coronavirus 2':ti,ab,kw OR sars:ti,ab,kw AND 'coronavirus 2':ti,ab,kw OR '2019 ncov':ti,ab,kw OR ('wuhan seafood market pneumonia virus':ti,ab,kw)) AND ('vascular endothelial growth factor*':ti,ab,kw OR vegf*:ti,ab,kw OR 'vascular endothelial growth factor a':ti,ab,kw OR 'vascular endothelial growth factor-a':ti,ab,kw OR 'vegf a':ti,ab,kw OR 'vascular permeability factor*':ti,ab,kw OR vascular:ti,ab,kw AND 'permeability factor*':ti,ab,kw OR vasculotropin:ti,ab,kw) | up to January 2024 | 619 |

**Supplemental Table2.**Quality assessment based on the Newcastle-Ottawa Scale of studies included in this meta-analysis.

| **Author, yrs.** | **Selection** | | | | **Comparability** | **Exposure** | | | **Score** |
| --- | --- | --- | --- | --- | --- | --- | --- | --- | --- |
|  | **An adequate definition of case** | **Representativeness of the case** | **Selection of controls** | **Definition of controls** | **cases and controls matched and/or adjusted by factors** | **Ascertainment of exposure** | **Same method** | **same response rate** |  |
| **Alfadda *et al.*, 2023** | ★ | ★ | ★ | ★ | ★ | ★ | ★ | – | 7 |
| **Mescht*et al.*, 2023** | ★ | ★ | – | ★ | ★ | – | ★ | – | 5 |
| ***Tsuji et al.*, 2023** | ★ | ★ | ★ | ★ | ★★ | – | ★ | ★ | 8 |
| **Josuttis *et al.*, 2023** | ★ | ★ | ★ | ★ | ★ | ★ | ★ | – | 7 |
| **Tufa *et al.*, 2022** | ★ | ★ | – | ★ | ★ | ★ | ★ | ★ | 7 |
| **Rovas *et al.*, 2021** | ★ | – | ★ | ★ | ★ | ★ | ★ | ★ | 7 |
| **Yazihan *et al.*,  2020** | ★ | ★ | ★ | ★ | ★★ | – | ★ | ★ | 8 |
| **Pine *et al.*, 2020** | ★ | ★ | – | ★ | ★ | ★ | ★ | – | 7 |
| **Smadja *et al.*, 2020** | ★ | ★ | ★ | ★ | ★ | – | ★ | – | 6 |
| **White *et al.*, 2020** | ★ | ★ | ★ | – | ★ | ★ | ★ | - | 6 |
| **Vassillou *et al.*, 2020** | ★ | ★ | – | ★ | ★★ | ★ | ★ | ★ | 8 |

**Supplemental Table 3.** The results of trim-and-fill method.


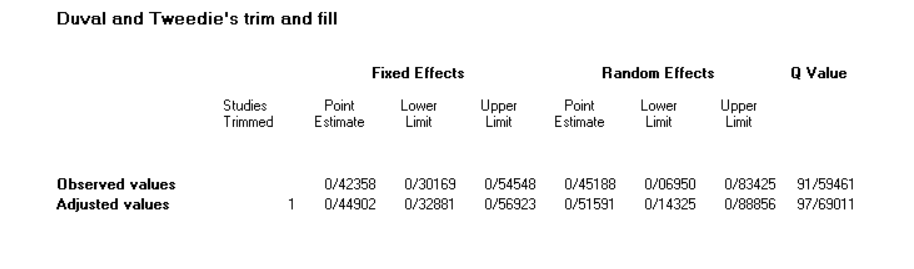


**Supplemental Table 4.** ROC analysis for VEGF-A levels as predictor of poor outcome in COVID‐19 patients.

| Author, year (Ref) | **AUC (95% CI)** | **Sensitivity % (95% CI)** | **Specificity% (95% CI)** | **Cutoff** | **P-value** |
| --- | --- | --- | --- | --- | --- |
| Alfadda 2023 | 0.9817(0.9703-0.9931) | 75.4(67.2-82.1) | 100(97.2- 100) | >22.50 | <.0001 |
| Tsuji 2023 | 0.68 [95%CI: 0.57-0.82] | 72%, | 65% | - | <.0001 |
| Josuttis 2023 | 0.734 [95%CI: 0.642-0.825] | 70.4% | 63.2%. | - | <.0001 |
| Tufa 2022 | 0.5059[0.4347- 0.5772] | 3.9(1.7- 8.9) | 97.8(93.6- 99.4) | <429.9 | .0868 |
| Rovas 2021 | 0.73 | - | - |  | <.0001 |
